# Angiotensin II-Induced Cardiovascular Fibrosis Is Attenuated by NO-Sensitive Guanylyl Cyclase1

**DOI:** 10.3390/cells9112436

**Published:** 2020-11-08

**Authors:** Kathrin Broekmans, Jan Giesen, Lukas Menges, Doris Koesling, Michael Russwurm

**Affiliations:** Institut für Pharmakologie und Toxikologie, Med. Fak. MA N1, Ruhr-Universität Bochum, 44780 Bochum, Germany; kathrin.broekmans@ruhr-uni-bochum.de (K.B.); jan.giesen@ruhr-uni-bochum.de (J.G.); lukas.menges@ruhr-uni-bochum.de (L.M.); doris.koesling@ruhr-uni-bochum.de (D.K.)

**Keywords:** angiotensin, nitric oxide, guanylyl cyclase, cGMP

## Abstract

In the NO/cGMP signaling cascade, relevant in the cardiovascular system, two NO-sensitive guanylyl cyclase (NO-GC) isoforms are responsible for NO-dependent cGMP generation. Here, the impact of the major NO-GC isoform, NO-GC1, on fibrosis development in the cardiovascular system was studied in NO-GC1-deficient mice treated with AngiotensinII (AngII), known to induce vascular and cardiac remodeling. Morphometric analysis of NO-GC1 KO’s aortae demonstrated an enhanced increase of perivascular area after AngII treatment accompanied by a higher aortic collagen1 mRNA content. Increased perivascular fibrosis also occurred in cardiac vessels of AngII-treated NO-GC1 KO mice. In line, AngII-induced interstitial fibrosis was 32% more pronounced in NO-GC1 KO than in WT myocardia associated with a higher cardiac Col1 and other fibrotic marker protein content. In sum, increased perivascular and cardiac interstitial fibrosis together with the enhanced collagen1 mRNA content in AngII-treated NO-GC1-deficient mice represent an exciting manifestation of antifibrotic properties of cGMP formed by NO-GC1, a finding with great pharmaco-therapeutic implications.

## 1. Introduction

cGMP is an important second messenger in the cardiovascular system and plays a crucial role in the regulation of smooth muscle tone. In addition, cGMP has been proposed to inhibit smooth muscle proliferation and to exert protective effects against cardiovascular remodeling and fibrosis [1,2,3]. Within the NO/cGMP pathway, the cGMP-forming, NO-sensitive guanylyl cyclase (NO-GC) acts as NO receptor. NO-GC is a heterodimeric protein composed of an α and a β subunit. Binding of NO to GC’s prosthetic heme-group stimulates cGMP production about 200-fold [4]. Two isoforms of NO-GC, NO-GC1 and NO-GC2, exist that share the common beta subunit (β1) but differ in the respective α subunit (NO-GC1 (α1β1) NO-GC2 (α2β1)) [5]. The two isoforms have indistinguishable catalytic and regulatory properties, but differ in their subcellular localization [6,7]. NO-GC1- and NO-GC2-deficient mice allow to analyze the function of the isoforms. The NO-GC1 isoform was identified as the major isoform in the vascular system [8]. Despite an NO-GC1 content of about 90%, NO-GC1 KO did not develop hypertension and revealed WT-like blood pressure increases upon application of the NO-synthase inhibitor L-NAME. In addition to NO-cGMP’s fundamental role in the regulation of smooth muscle tone, antifibrotic properties of NO-dependent cGMP have been proposed to occur in various organs including in the cardiovascular system [2].

Recently, measurements of NO-induced cGMP signals in cardiac cells revealed NO-GC-dependent cGMP to be exclusively produced in cardiac fibroblasts whereas in cardiac myocytes only the natriuretic peptide CNP induces cGMP production [9]. The occurrence of NO-induced cGMP in cardiac fibroblasts prompted us to speculate about a possible role of cGMP in fibrosis. The contractile agonist Angiotensin II (AngII), the most important messenger of the renin-angiotensin-system, is known to cause hypertrophy and fibrosis in blood vessels and to stimulate proliferation of fibroblasts, expression of extracellular matrix proteins, and hypertrophy of myocytes within cardiac remodeling [10,11,12].

Here, a possible role of NO-induced cGMP in fibrosis development was addressed by treating mice deficient in the major NO-GC isoform NO-GC1 with an AngII known to cause fibrosis. AngII-induced blood pressure increases in NO-GC1 and WT were comparable. With concern to fibrosis, perivascular areas of aortae of AngII-treated NO-GC1 KOs were augmented as was the aortic collagen 1 mRNA content. Moreover and most importantly, cardiac perivascular and interstitial fibrosis of AngII-treated NO-GC1 KOs was more pronounced than in WT. The increased AngII-induced cardiac fibrosis in the NO-GC1 KOs was accompanied by increased mRNA content of key mediators of cardiac fibrosis (collagen 1, Tgfβ, and periostin). Thus, increased cardiac fibrosis in AngII-treated NO-GC1-deficient mice impressively demonstrated antifibrotic properties of cGMP in vivo.

## 2. Materials and Methods

### 2.1. Animal Models

NO-GC1 KO mice were generated as described [8]. Eight to twelve-week-old male NO-GC1 KO mice (backcrossed to C57Bl/6Rj) and WT littermates were infused with AngII (1.44 mg/kg/d) using osmotic mini pumps (model 1002; Alzet) for two weeks as described [13] according to European and German law and as approved by local authorities (Landesamt für Natur, Umwelt und Verbraucherschutz Nordrhein-Westfalen, 84-02.04.2012.A353). Subsequent to the AngII treatment, mice were sacrificed and used for following analyses.

### 2.2. Morphometric Analysis of Aorta

Abdominal aortae between the left and right renal arteries were fixed in 4% Rotifix (Carl Roth, Kalrsruhe, Germany; 48 h) and embedded in paraffin. Transversal sections (5 µm) were stained with picrosirius red (0.1%; 60 min) and documented (VS120, Olympus, Hamburg, Germany). Using ImageJ [14], medial area was measured as area between the external and internal elastic laminae; wall thickness was determined as difference between medial and luminal radii derived from luminal and medial areas of (almost) circular sections; and perivascular area was measured as dark-stained area (picrosirius red) surrounding the vessel. Elastin as stained with elastica Van Gieson was documented and quantified as above and normalized to medial area. Three sections per animal were quantified by a blinded investigator.

### 2.3. Histological Analysis of Cardiac Slices

Freshly prepared hearts were fixed, embedded, cut, and stained as above. Perivascular and medial areas of cardiac vessels were measured as above. Interstitial fibrosis was quantified at a distance of ≈1.5 and ≈2 mm from the apex in three sections/animal as percentage of picrosirius red-stained area in ten random fields by a blinded operator using ImageJ.

WGA staining was performed using fluorescein-coupled wheat germ agglutinin (Vector Labs., Burlingame, CA, USA). Images were recorded on a Nikon (Amsterdam, Netherlands) Eclipse Ti-E inverted confocal microscope and cardiomyocyte cross sectional areas were analyzed by a blinded operator using ImageJ.

### 2.4. RNA Isolation, cDNA Synthesis and Quantitative Real Time PCR

Aortic RNA was isolated using NucleoSpin Triprep kit (Macherey-Nagel, Düren, Germany) according to the manufacturer’s instructions (homogenization in 350 µL RP1, glass/glass Potter-Elvehjem, 900 rpm; elution 40 µL). RNA from paraffin-embedded heart tissue was extracted using NucleoSpin total RNA FFPE kit (Macherey-Nagel) according to the manufacturer’s instructions (15 sections à 30 µm; elution 30 µL).

Total RNA (8 µL) was reverse transcribed using the SuperScript II first strand synthesis system for RT PCR (Invitrogen, Schwerte, Germany) following the manufacturer’s instruction. Real-time PCR was performed with the Blue S’Green qPCR Kit (Biozym, Hessisch Oldendorf, Germany) on a GeneAmp 5700 Sequence Detection system (Perkin Elmer, Rodgau, Germany) using the following primers: GAPDH, AACTTTGGCATTGTGGAAGG, CACATTGGGGGTAGGAACAC; Collagen1, CATGTTCAGCTTTGTGGACCT, GCAGCTGACTTCAGGGATGT; Collagen3, CACAATATAAGAGGCATGACAGACG, TCCAGGAGCACCGACCTT; FN, TTGTTCGGTGGAGTAGACCC, GTGCCAGTGGTCTCTTGTTG; CTGF, TGACCTTGGAGGAAAACATTAAGAA, AGCCCTGTATGTCTTCACACTG; Pstn, AACCAAGCACCTGAAACACG, TGTGTCAGGACACGGTCAAT; ANP, GAAGATGCCGGTAGAAGATG, CTCTCAGAGGTGGGTTGA; BNP, GGGAGGCGAGACAAGGGAGAAC, CAGCGGTGACAGATAAAGGAAAAG; MMP2, CCTTCACTTTCCTGGGCAAC, GGTGTAGGTGTAGATCGGGG; TGFβ, TGGAGCAACATGTGGAACTC, CAGCAGCCGGTTACCAAG. Samples were measured in triplicates; samples of non-treated and AngII-treated WT and NO-GC1 KO mice were analyzed in parallel for each gene. Ct values of triplicates obtained from the GeneAmp software were averaged and normalized to GAPDH Ct values (ΔCT) and subsequently to values obtained for non-treated WT (ΔΔCT).

### 2.5. Statistical Analysis

Besides the experiment reporting cGMP-forming activities (analyzed using unpaired two-sided *t* test), all other data sets were analyzed by one-way ANOVA with Sidak’s multiple comparisons test (WT non-treated vs. WT AngII-treated; NO-GC1 KO non-treated vs. NO-GC1 AngII-treated; WT non-treated vs. NO-GC1 KO non-treated; WT AngII-treated vs. NO-GC1 AngII-treated) to obtain multiplicity-adjusted *p* values and 95% confidence intervals (Prism 8.3.1, Graphpad, San Diego, CA, USA). As sample sizes had been adapted during the experiment, *p* values and confidence intervals should be considered descriptive and not as hypothesis-testing. In the results section, effect sizes and 95% confidence intervals (given in parentheses) are reported as percent changes and *p* values are given in the figures. For sake of clarity, some (high) *p* values are omitted.

## 3. Results

Of the two NO-GC isoforms, NO-GC1 has been identified as the major NO-GC isoform in the vascular system. Despite only 10% of NO-sensitive cGMP-forming activity being left in the vasculature of NO-GC1 KOs, the mice were not hypertensive [8]. Here, NO-GC1 KOs and the respective WT mice were subjected to a chronic AngII treatment (high dose, 1.44 mg/kg per day, over 2 weeks) known to induce vascular and cardiac remodeling and fibrosis. The blood pressure was increased by AngII treatment; however, AngII-induced blood pressure increases did not differ between NO-GC1 KOs and WT [12]. In the following, AngII-induced vascular changes were assessed subsequent to the treatment.

### 3.1. Enhanced AngII-Induced Perivascular Fibrosis in Aortae of NO-GC1 KOs

**Figure 1:**
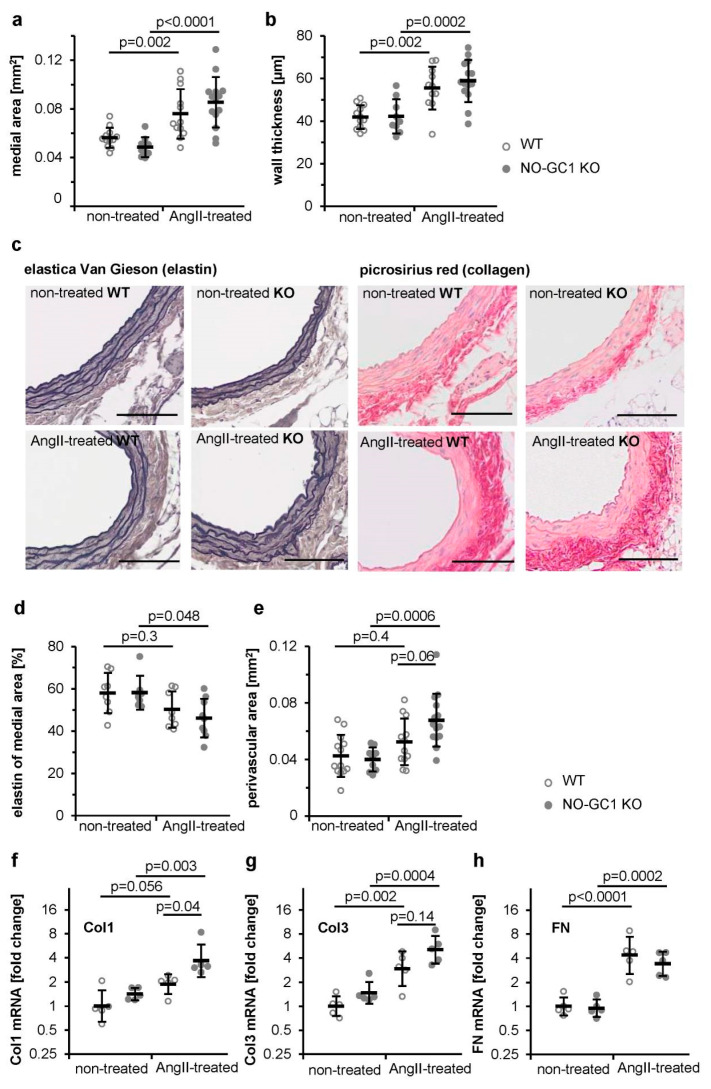
In NO-sensitive guanylyl cyclase (NO-GC)-1 KO mice, perivascular fibrosis and collagen1 mRNA content were increased by AngiotensinII (AngII) treatment. Medial area (**a**) and wall thickness (**b**) were measured in cross sections (5 µm) of abdominal aortae from non-treated and AngII-treated WT and NO-GC1 KO mice. Shown are individual values (per animal) together with means ± SD. (**c**) Representative cross sections (5 µm) of abdominal aortae from non-treated and AngII-treated WT and NO-GC1 KO mice stained with elastica Van Gieson (for elastin) and picrosirius red (for collagen). Scale bar, 100 µm. (**d**) Elastin content is expressed as percentage of medial area of aortae from non-treated and AngII-treated WT and NO-GC1 KO mice. (**e**) Perivascular area of aortae from non-treated and AngII-treated WT and NO-GC1 KO mice were quantified. Shown are individual values (per animal) together with means ± SD. mRNA levels of (**f**) collagen1 (**g**) collagen3, and (**h**) fibronectin (FN) were determined using quantitative real-time PCR. ∆∆Ct values to GAPDH and subsequently to non-treated WT were calculated and statistically analyzed. For graphical representation, 2^−∆∆C^ are shown in semi-log plots. Shown are individual values (per animal) together with geometric means ± geometric SD.

Vascular remodeling comprises changes of vessel geometry and extracellular matrix composition. Therefore, we analyzed aortic cross sections of WT and NO-GC1 KO mice with or without AngII-treatment. Aortic medial areas measured as a parameter for vascular remodeling of WT and NO-GC-1 KO mice were indistinguishable under non-treated conditions (Figure 1a); AngII treatment increased the medial areas comparably in both genotypes in WT and NO-GC1 KO, respectively. Likewise, the approximate 30% increase of the aortic wall thickness induced by AngII was similar in both mice strains (Figure 1b).

Increased extracellular matrix (ECM) production in the course of AngII-induced vascular remodeling is well established. In accordance, the ECM composition of the extracellular matrix was determined by histological staining of aortic cross sections (Figure 1c). Additionally, the elastin content of aortae was determined by elastica Van Gieson staining. In aortae of non-treated mice, the elastin content of the media was comparable. However, in NO-GC1 KOs only, AngII treatment decreased the elastin content (by 21%, 95% confidence interval 0.2–41%) (Figure 1d). By analysis of picrosirius red staining, the collagen content in the perivascular area of the aorta was quantified. In non-treated mice, perivascular collagen was comparable (Figure 1e). AngII treatment increased aortic perivascular collagen only in NO-GC1 KOs by ≈70% (26–112%) resulting in a higher collagen content in NO-GC1 KOs than in WT. Enhanced perivascular collagen deposition in the NO-GC1 KO strain is indicative of an antifibrotic effect of cGMP. To verify the result, we determined the mRNA content of components of the extracellular matrix (collagen 1, 3, and fibronectin) in aortae of non-treated and AngII-treated mice. In non-treated mice, aortic mRNAs tested did not differ between WT and NO-GC1 KOs (Figure 1f–h). Whereas the AngII-induced increase of the mRNA content of fibronectin and collagen 3 was similar in both genotypes, in NO-GC1 KO the aortic collagen 1 mRNA content was increased by AngII (by 160%, 36–400%) resulting in twice as high collagen 1 mRNA levels in NO-GC1 KO compared to WT. The results are in accordance with the increased perivascular fibrosis and indicate an inhibitory effect of NO-GC1 on perivascular collagen synthesis.

### 3.2. Enhanced AngII-Induced Cardiac Perivascular and Interstitial Fibrosis in NO-GC1 KOs

**Figure 2:**
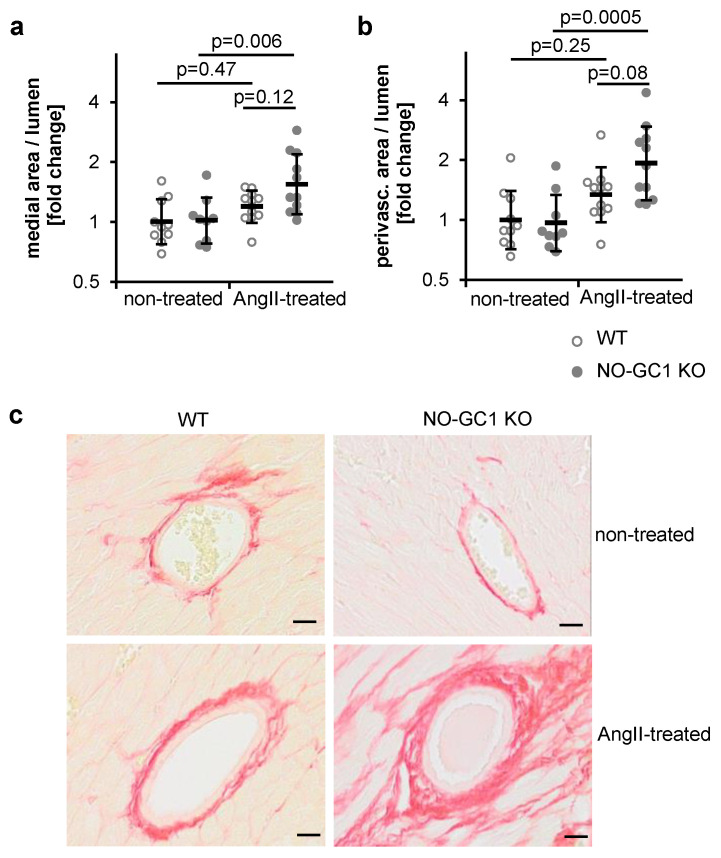
AngII induces an increase of perivascular area of cardiac vessels in NO-GC1 KO mice. (**a**) Media to lumen and (**b**) perivascular to lumen ratios of cardiac vessels were determined in picrosirius red-stained heart slices from non-treated and AngII-treated WT and NO-GC1 KO mice. Values are normalized to media to lumen ratio and perivascular area to lumen ratio of WT (in a: 0.59; in b: 0.53). Because residuals were not normally distributed, individual values were log-transformed and statistically analyzed. For graphical representation, original (not-transformed) values are shown in semi-log plots as individual values (per animal) together with geometric means ± geometric SD. (**c**) Representative cross sections (5 µm) of cardiac vessels from non-treated and AngII-treated WT and NO-GC1 KO mice stained with picrosirius red (for collagen). Scale bar, 20 µm.

Next, we studied cardiac vessels by picrosirius red staining of heart sections and quantified vascular medial areas and perivascular fibrosis. In non-treated mice, the media to lumen ratios were indistinguishable (Figure 2a). In the NO-GC1 KOs only, AngII increased the media (by 52%, 10–109%). The perivascular area of cardiac vessels did not differ between WT and NO-GC1 KOs under non-treated conditions (Figure 2b). Analogous to the medial areas, AngII increased the perivascular collagen content in NO-GC1 KOs by 99% (31–203%) but not in WT (−11–101%, Figure 1c). The enhanced AngII-induced perivascular collagen deposition in the NO-GC1 KOs is in accordance with the augmented perivascular collagen deposition in the aortae.

**Figure 3:**
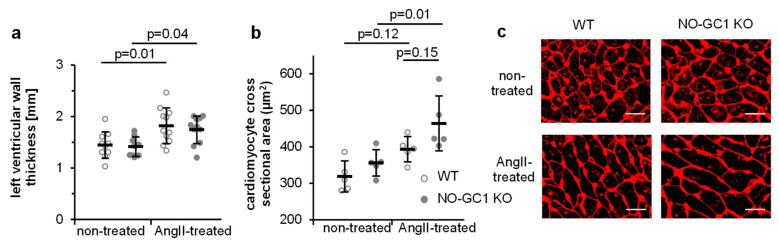
AngII induced increase of left ventricular wall thickness and cardiomyocyte cross sectional area. (**a**) Left ventricular wall thickness was measured in hearts of non-treated and AngII-treated WT and NO-GC1 KO mice. Individual values (per animal) are shown together with means ± SD. (**b**) Cardiomyocyte cross sectional areas of WGA-stained heart slices (**c**) were quantified. Individual values (per animal) are shown together with means ± SD. (**c**) Shown are representative WGA-stained heart sections. Scale bar, 20 µm.

To determine the cardiac NO-GC isoform composition, NO-stimulated cGMP-forming activity was measured in the heart homogenates. As in blood vessels, NO-GC1 turned out to be responsible for the vast majority of the NO-dependent cGMP formation (WT 1.6 ± 0.4; KO 0.24 ± 0.05 nmol/min/mg, *p* = 0.0003). Similarly, NO-stimulated cGMP forming activity in the homogenate of cardiac fibroblasts was found to be greatly reduced in the NO-GC1 KO (WT 1.8 ± 0.12; KO 0.32 ± 0.07 nmol/mg/min), confirming NO-GC1’s major role in cardiac fibroblasts. Thus, NO-GC1 KO mice should reveal the impact of reduced NO-dependent cGMP on AngII-induced cardiac hypertrophy and fibrosis. Cardiac hypertrophy (assessed as heart to body weight ratio) induced by AngII was similar in both strains as has been shown before [13]. Likewise, wall thickness of the left ventricle, determined as an additional parameter for cardiac hypertrophy, did not differ between WT and NO-GC1 KO hearts under non-treated and AngII-treated conditions (Figure 3a). Cardiomyocyte cross sectional areas assessed by WGA staining revealed an increase of cardiomyocyte size by AngII in NO-GC1 KO mice (Figure 3b).

**Figure 4:**
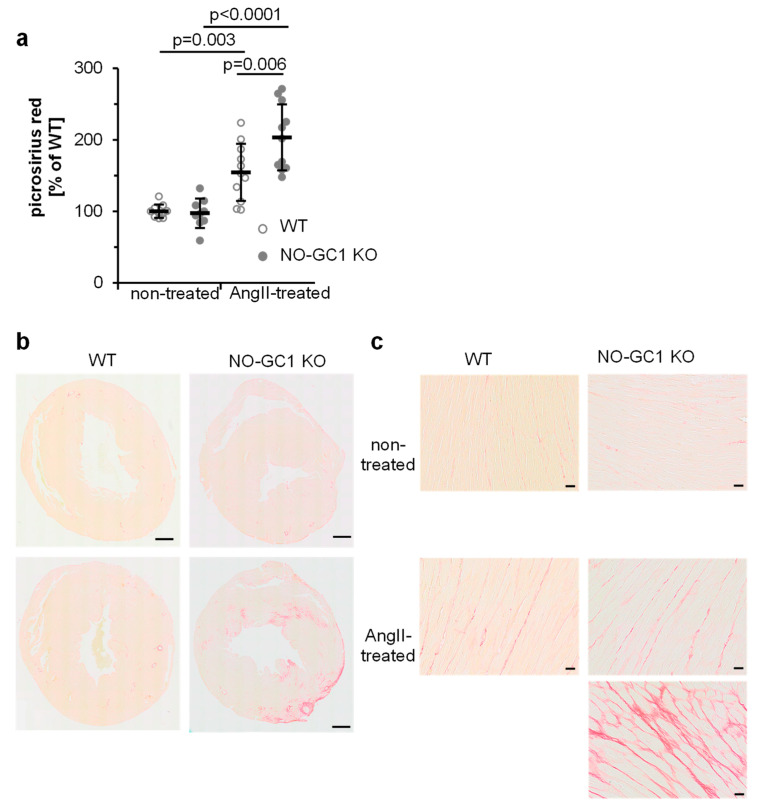
NO-GC-1 KOs display substantially enhanced AngII-induced interstitial fibrosis. (**a**) Picrosirius red-stained areas of hearts (**b**,**c**) were quantified and normalized to non-treated WT. Shown are individual values (per animal) together with means ± SD. (**b**) Shown are representative paraffin-embedded heart sections (5 µm) stained with picrosirius red. Scale bar, 500 µm (**c**) Shown are magnifications of heart sections stained with picrosirius red. Strongly red stained areas found in NO-GC1 KO hearts were not used for analysis. Scale bar, 20 µm.

To determine cardiac interstitial fibrosis, we quantified (Figure 4a) picrosirius red-stained areas of cardiac sections (Figure 4b,c). While under non-treated conditions stained areas did not differ between WT and NO-GC1 KOs hearts (see Figure 4a), interstitial fibrosis induced by AngII increased by 109% (69–150%) in NO-GC1 KO compared to 54% (16–93%) in WT. Thus, interstitial fibrosis is enhanced by 32% in NO-GC1 deficient mice (7–56%). The blinded investigator did not quantify intensely stained areas found in the NO-GC1 heart sections after AngII treatment (see Figure 4b,c). These results underline the strong impact of cGMP on cardiac collagen synthesis and fibrosis.

**Figure 5:**
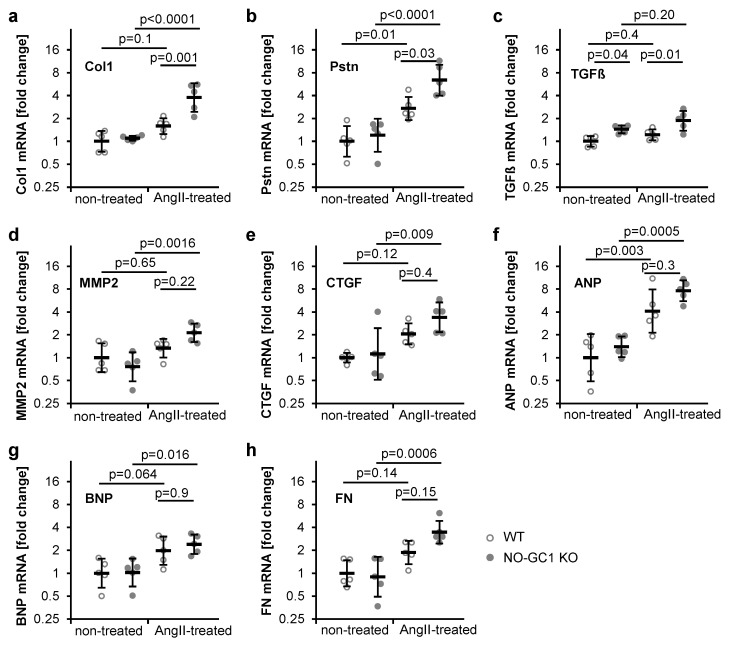
Cardiac mRNA of proteins implicated in fibrosis are increased in AngII-treated NO-GC1 KO compared to WT mice. mRNA levels of (**a**) collagen 1 (**b**) periostin (**c**) TGFβ (**d**) matrix metalloproteinase 2 (**e**) CTGF (**f**) ANP (**g**) BNP, and (**h**) fibronectin were determined using quantitative real-time PCR in heart sections from WT and KO mice under non-treated and AngII-treated conditions. ∆∆Ct values to GAPDH and subsequently to non-treated WT were calculated and statistically analyzed. For graphical representation, 2^−∆∆Ct^ values were calculated and are shown in semi-log plots. Shown are individual values (per animal) together with geometric means ± geometric SD.

To confirm the findings, the expression of proteins implicated in cardiac fibrosis was measured by quantitative real-time PCR. ANP mRNA levels were similarly increased by the AngII treatment in WT and NO-GC1 KO mice (Figure 5f). Only the NO-GC1 KOs displayed AngII-induced increases of the fibrotic markers MMP2, CTGF, BNP, and FN (Figure 5d,e,g,h). Moreover, AngII increased Col1 mRNA only in the NO-GC1 KO (250%, 104–480%) leading to 138% (41–303%) higher Col1 levels in the NO-GC1 KO compared to WT (Figure 5a). Similarly, the AngII-induced cardiac periostin mRNA contents were 137% (6–430%) higher in NO-GC1 KOs than in WT (Figure 5b). Furthermore, the TGFβ mRNA content of NO-GC1 KOs was higher than in WT under non-treated (44%, 2–104%) and AngII-treated conditions (53%, 8–117%, Figure 5c). These findings corroborate the histological data obtained in cardiac slices and are in accordance with an inhibitory effect of NO-induced cGMP on collagen production and cardiac fibrosis.

## 4. Discussion

In the present study, the profibrotic impact of NO-GC1 deficiency upon AngII challenge was studied. The blood pressure increases induced by the AngII treatment were comparable in WT and NO-GC1 KOs. Conceivably, this is due to the previously identified reduction of sympathetic output indicated by the lower plasma noradrenalin content in NO-GC1 KO mice under non-treated and AngII-treated conditions, which is also underlined by the reduction of the heart rate [8,13]. Thus, the differential development of fibrosis in WT and NO-GC1 KOs can be regarded as blood pressure independent.

Whereas NO/cGMP-induced vasorelaxation as prototypical biological NO response has been known for decades, the protective role of NO-induced cGMP in cardiovascular events such as cardiovascular remodeling and fibrosis is a matter of current research [1,3]. A possible role of NO-dependent cGMP in fibrosis development is further underlined by the finding of comparably high NO-induced cGMP signals in cardiac fibroblasts and their recently described absence in cardiac myocytes [9]. As AngII induces cardiovascular remodeling/fibrosis, NO-GC1 KOs challenged by AngII treatment were analyzed to verify the protective role of NO/cGMP in these events.

Hypertension and cardiac hypertrophy induced by AngII treatment did not differ between NO-GC1 KOs and WT mice [13]. In addition, aortic medial areas and aortic wall thickness, analyzed as parameters for vascular remodeling, increased upon AngII treatment; however, neither of these factors revealed differential responses of NO-GC1 KO compared to WT mice. In contrast, the AngII-induced aortic perivascular collagen deposition was higher in NO-GC1 KOs. The conclusion of a protective role of cGMP in perivascular fibrosis is further strengthened by higher aortic Col1 mRNA content and the increased cardiac perivascular areas in AngII-treated NO-GC1 KOs.

Finally, while cardiac collagen content did not differ under baseline conditions, interstitial fibrosis induced by AngII treatment emerged to be more pronounced in the NO-GC1 KOs. The notion of aggravation of AngII-induced interstitial fibrosis under conditions of low NO-dependent cGMP is supported by the higher cardiac Col1 mRNA content in AngII-treated NO-GC1 KOs. Moreover, key mediators of cardiac fibrosis, periostin and TGFβ, were increased in AngII-treated NO-GC1 KOs compared to WT, with the TGFβ mRNA being higher in the NO-GC1 KOs already under non-treated conditions.

In sum, the perivascular collagen content of aortae and cardiac vessels as well as cardiac interstitial fibrosis induced by AngII treatment were found to be enhanced by NO-GC1 deficiency. These findings indicate an inhibitory effect of NO-induced cGMP on extracellular matrix production and cardiac fibrosis and bear great pharmaco-therapeutical implications. In accordance with our results, eNOS KOs or inhibition of NOS activity resulted in enhanced cardiac fibrosis induced by transverse aortic constriction [15]. Pharmacologically most interesting and in support of our results are the findings that cardiac perivascular and interstitial fibrosis induced by aortic constriction or AngII treatment in rats were (partially) prevented by an NO sensitiser/NO-GC stimulator [16,17]. In mice, the PDE5 inhibitor sildenafil was reported to decrease AngII-induced cardiac fibrosis in the left ventricle [18], an effect depending on the presence of cGMP-dependent protein kinase in cardiac cells (specifically, non-smooth muscle cells). In accordance with a general antifibrotic cGMP effect, ANP and BNP inhibited cardiac fibrosis as well [19,20,21]. The principle of increasing natriuretic peptides is already applied with Sacubitril, in which a vasopeptidase (Neprilysin, degrades i.a. ANP and BNP) inhibitor is combined with an AT1-receptor inhibitor. Sacubitril is approved for treatment of heart failure.

Recently, a clinical trial with an NO sensitiser/NO-GC stimulator in the treatment of heart failure with reduced ejection fraction has been completed [22]. Patients receiving the NO-GC stimulator vericiguat displayed a lower incidence of death from cardiovascular causes or hospitalization for heart failure than those receiving a placebo. Soon, the results of another trial with the NO-GC stimulator in the treatment of heart failure with preserved ejection fraction, HFpEF, will be available and show whether NO-GC stimulation exerts beneficial effects in this special form of heart failure which so far has been difficult to pharmacologically address [23].

While the occurrence of NO-induced cGMP signals in cardiac cells has been a matter of debate [24,25,26,27,28], a recent publication revealed pronounced NO-induced cGMP responses in cardiac fibroblasts but none in isolated cardiac myocytes [9]. The occurrence of NO-GC in cardiac fibroblasts is compatible with an antifibrotic action. Together with the data obtained here, the results assign a role to NO-GC1 in limiting fibrosis and propose NO-GC as a promising drug target to prevent cardiac fibrosis within the development of heart failure.
